# Intervention fidelity in a school-based diet and physical activity intervention in the UK: Active for Life Year 5

**DOI:** 10.1186/s12966-015-0300-7

**Published:** 2015-11-11

**Authors:** Rona Campbell, Emma Rawlins, Sian Wells, Ruth R. Kipping, Catherine R. Chittleborough, Tim J. Peters, Debbie A. Lawlor, Russell Jago

**Affiliations:** School of Social and Community Medicine, University of Bristol, Bristol, UK; School of Population Health, University of Adelaide, Adelaide, Australia; School of Clinical Sciences, University of Bristol, Bristol, UK; MRC Integrative Epidemiology Unit at the University of Bristol, Bristol, UK; Centre for Exercise, Nutrition & Health Sciences, School for Policy Studies, University of Bristol, Bristol, UK

**Keywords:** RCT, School-based intervention, Process evaluation, Fidelity, Mixed methods, Diet, Physical activity, UK

## Abstract

**Background:**

Active for Life Year 5 (AFLY5) is an educational programme for Year 5 children (aged 9–10) designed to increase children’s physical activity, decrease sedentary behaviour and increase fruit and vegetable intake. This paper reports findings from a process evaluation embedded within a randomised controlled trial evaluating the programme’s effectiveness. It considers the fidelity of implementation of AFLY5 with a focus on three research questions:

1. To what extent was the intervention delivered as planned?
2. In what ways, if any, did the teachers amend the programme? and
3. What were the reasons for any amendments?

**Methods:**

Mixed methods were used including data collection via observation of the intervention delivery, questionnaire, teacher’s intervention delivery log and semi-structured interviews with teachers and parents. Qualitative data were analysed thematically and quantitative data were summarised using descriptive statistics.

**Results:**

Following training, 42 of the 43 intervention school teachers/teaching staff (98 %) were confident they could deliver the nutrition and physical activity lessons according to plan. The mean number of lessons taught was 12.3 (s.d. 3.7), equating to 77 % of the intervention. Reach was high with 95 % of children in intervention schools receiving lessons. A mean of 6.2 (s.d. 2.6) out of 10 homeworks were delivered. Median lesson preparation time was 10 min (IQR 10–20) and 28 % of lessons were reported as having been amended. Qualitative findings revealed that those who amended the lessons did so to differentiate for student ability, update them for use with new technologies and to enhance teacher and student engagement. Teachers endorsed the aims of the intervention, but some were frustrated with having to adapt the lesson materials. Teachers also a reported tendency to delegate the physical activity lessons to other staff not trained in the intervention.

**Conclusions:**

Fidelity of intervention implementation was good but teachers’ enthusiasm for the AFLY5 programme was mixed despite them believing that the messages behind the lessons were important. This may have meant that the intervention messages were not delivered as anticipated and explain why the intervention was found not to be effective.

**Trial registration:**

ISRCTN50133740.

## Background

Physical activity has been associated with lower levels of cardio-metabolic risk factors, improved mental well-being and a lower risk of obesity in young people [1]. Fruit and vegetable consumption is associated with lower caloric intake and a reduction in the risk of many forms of cancer and heart disease among adults [2–7], with patterns of consumption being established in childhood [4, 8]. Many young people in the UK do not meet the current recommendation of an hour of physical activity on most days of the week [9, 10] and do not consume the recommended 5 fruits and vegetables per day [11]. Developing strategies to increase young people’s physical activity and fruit and vegetable consumption and decrease sedentary behaviour is an important public health priority.

Schools provide opportunities to reach the majority of children and as such have enormous potential to provide physical activity and nutrition focussed public health interventions [12]. Recent school-based interventions designed to improve diet and physical activity and decrease sedentary behaviour have reported only modest positive outcomes [3, 13–15] so more effective interventions are required. When designing new interventions it is important to learn from previous successes and failures [16–18] and assessing intervention fidelity is a key component of this process.

Implementation fidelity is the extent to which an intervention is delivered as expected [18–23]. Assessing fidelity [19, 20, 22, 24–26] can help determine: whether and how variations in delivery occurred [19, 23]; whether or not the intervention was likely to be effective [19, 22]; and how variations in delivery may have affected intervention outcomes [19, 23]. Once fidelity has been established the sustainability of the intervention can be considered. An important aspect of sustainability is the potential level of fidelity of implementation when the intervention is carried out in a ‘real world’ setting [22, 24]. Thus, in order to assess the sustainability of the intervention it is necessary to consider how variations in delivery occurred and identify aspects of the intervention that can be modified without compromising the underpinning theory and ‘spirit’ of the intervention or having an adverse impact on effectiveness.

Active for Life Year 5 (AFLY5) was a cluster randomised controlled trial in state primary schools designed to increase physical activity and fruit and vegetable intake while also decreasing sedentary time. The AFLY5 intervention comprised of training for Year 5 teachers and Learning Support Assistants on how to teach the intervention which consisted of 16 lesson plans, 10 homeworks in which the children were encouraged to work with their parent, two parental information leaflets, and inserts for school newsletters. Table 1 lists the intervention elements, resources provided and the delivery timetable. Control schools were offered the opportunity to receive the training and intervention materials after the final follow-up measurements were taken in the summer of 2013.

**Table 1:** AFLY5 Intervention elements

Intervention elements	Resources provided	Delivery	Timeframe
Teacher training	Refunded travel expenses	1 day long	September 2011 to January 2012
	Refunded cost of supply teacher.	Choice of 5 dates	
Lessons	Folder containing: 1) overview and lesson plans for 16 lessons: **·**10 physical activity focussed, 6 nutrition focussed **·**lasting 30–60 min 2) Paper copies of worksheets for each child 3) CD with further information and back-up copies of materials	Teachers were strongly encouraged to deliver all 16 lessons. 8 lessons were identified as particularly important and teachers were told that if short of time these 8 lessons should be prioritised	From teacher training date until July 2012
		Timing determined by teachers	
Homework	Paper copies of 10 homeworks for each child	Homeworks were paired with specific lessons, but teachers determine timing of delivery	From training date until May 2012
	Back-up copies on CD		
Hand out information leaflets for parents	Paper copies of 2 leaflets for each child: “Top Tips for Top Kids” “Freeze my TV tips for parents and guardians”	Teachers determine timing of delivery	From training date until May 2012
Snippets to be added to school newsletters	6 snippets provided on CD	Teachers determine timing of delivery	From training date until May 2012

The effectiveness evaluation of AFLY5 found that there was no difference in the primary outcomes of accelerometer measured physical activity, sedentary time or self-reported fruit and vegetable consumption among children in intervention compared to control schools, though there were beneficial effects with respect to reducing self-reported consumption of high energy drinks, snacks and screen-viewing time [27].

This paper reports findings from a process evaluation embedded within the randomised controlled trial evaluating the programme’s effectiveness. It considers the fidelity of implementation of AFLY5 with a focus on three research questions:

1. To what extent was the intervention delivered as planned?
2. In what ways, if any, did the teachers amend the programme? and
3. What were the reasons for any amendments?

Findings from the process evaluation not included in this paper are reported elsewhere, Lawlor D, Kipping R, Anderson E, Howe L, Chittleborough C, Moure-Fernandez A, Noble S, Rawlins E, Wells S, Mytton J, et al: Active For Life Year 5: A cluster randomised controlled trial of a primary school-based intervention to increase levels of physical activity, decrease sedentary behaviour and improve diet. Forthcoming [28].

## Methods

The AFLY5 trial was conducted in Bristol and North Somerset in England and ran from May 2011 to July 2013 [27]. The intervention was delivered from September 2011 to July 2012 to children in Year 5 (aged 9–10). Sixty schools took part, with 30 schools randomly allocated to the intervention and 30 to the control arm. One school randomised to the intervention arm later decided not to deliver the intervention but agreed to participate in trial data collection. All children in both the intervention and control schools took part in a series of measurements at baseline (Year 4), post intervention (Year 5) and at follow-up one year later (Year 6) (see the trial protocol [29] and statistical analysis plan [30] for more details).

Process evaluation data were collected at three different phases of the project. Phase one started before the intervention began; phase two ran during the intervention; and phase three began once the intervention had ended. Details of the number of participants in each of these phases, and the methods used to collect and analyse the data are provided below and summarised in Table 2. Data were collected to evaluate the fidelity of intervention delivery, explore whether teacher, parent and child responses were consistent with how the intervention was theorised to act in our logic model and any potential barriers to wider dissemination should it prove effective.

**Table 2:** Details of data collected and analysed to assess and understand fidelity of intervention delivery

Method of data collection	Data collected from or by	Number completed	Response rate	Data collection timeframe	Data collected	Data collection format
Phase 1						
Teacher training observations	Observing 43 teachers from 29 intervention schools and 2 trainers. (44 teachers were invited to the training but one was unable to attend.)	*n* = 5	100 % of sessions observed	During training sessions	Details on venue, number of trainers present, number of participants, gender	Pro forma layout
				Sessions ran September 2011- January 2012	Delivery and content of training	Free form text
					Teacher engagement and understanding	Free form text
					Questions or issues raised by teachers	Free form text
					Detailed description of activities	Free form text
					Reflection on the observation process	Free form text
Teacher training evaluation questionnaire	Teachers	*n* = 43	100 %	Completed at the end of training session	Whether they felt confident that they had enough knowledge to teach the nutrition and physical activity sessions successfully	5 point scale: strongly agree, agree, don’t know, disagree, strongly disagree
					Whether they felt confident to teach the lessons according to the plans	5 point scale as above
					Whether they felt they needed more information in order to teach the lessons	5 point scale as above
					Whether they were confident at fitting all 16 lessons into the allotted time frame	5 point scale as above
					Indicating 3 key messages of AFLY5	Free text
					Indicating how useful the day was in terms of preparing for AFLY5	3 points: Very useful, useful in places, not useful
						Space for comments if selected ‘not useful’
Phase 2						
Lesson observations	Observing 30 lessons being taught in 24 of the 29 schools delivering AFLY5	*n* = 30	24 out of 30 (80 %) schools had at least one lesson observed	November 2011 to April 2012	Details of lessons including: number of children, gender, teacher identity code	Pro forma layout
					Observation of the general behaviour of children	3 point scale: good, acceptable, poor
					Observation of the level of interest and enthusiasm displayed by the children	3 point scale: high, indifferent, low
			15/16 (94 %) lessons observed at least once			
					Whether delivery of key outcomes of lesson were met	Yes/no
					Whether resources provided were used	Yes/no
					Whether homework was handed out	Yes/no
			11/16 (69 %) observed twice			
					More detailed notes including: **·**layout of room **·**children’s behaviour and engagement **·**suitability of content for the ability of the group **·**aspects of the lesson that worked well/less well	Free form text
Teacher intervention delivery logs	44 teachers in the 29 schools delivering AFLY5	*n* = 44	100 % for data on total number of lessons delivered	September 2011- July 2012	Teachers involved with delivery of AFLY5	Pro forma table
					Their position in the school (e.g. Newly Qualified, Support Staff, Managerial Scale etc.)	
			39 out of 44 teachers (89 %) provided partial data on all other aspects of log			
					Whether they attended the AFLY5 training	Yes/no
					Information requested per lesson: **·**Who taught the lesson **·**Date taught **·**Number of children present	Pro forma table
					Amount of time spent preparing for the lesson	Pro forma table recorded in minutes
					Amount of time spent delivering the lesson	
					Whether any other resources were required.	Yes/no options
					If so, what and how much did they cost	Pro forma table recorded in pounds
					Whether there were any difficulties with the lesson	Yes/no
					If so, what	Free form text
					Whether any amendments were required	Yes/no
					If so, what	Free form text
					What they would have been teaching instead of AFLY5	Free form text
					Who would have led this lesson	Free form text
					Whether there was more or less preparation for AFLY5 than usual lessons	3 point scale: more, less, same
					Whether the homeworks were handed out	Yes/no
					How many were completed	Free form text
					The quality of homeworks	3 point scale: good, fair, poor
					**·**teacher’s understanding of subject area **·**delivery style of teacher **·**input from other staff members **·**if the lesson was taught according to the plan **·**reflections on the research process	
					Any extra comments on both a per lesson basis, as well as at the end of the log	Free form text
Phase 3						
Interviews	20 teachers from 15 of the 29 schools delivering AFLY5	*n* = 20	20 out of 44 teachers (45 %)	October 2012 to April 2013	Semi structured interview questions on the following topics: **·**What contributes to a healthy lifestyle both generally and for children **·**Teaching health promotion in schools **·**Whether they were involved with any health promotion projects **·**Whether school-based health promotion education is effective in changing children’s behaviour *Their experience of teaching AFLY5	Audio recording/transcript
Teachers						
		Face to face = 14/20 (70 %)				
		Mean length of interview = 32 min				
		Range = 17–57 min				
		Phone = 6/20 (30 %)				
		Mean length of interview = 35 min				
		Range = 28–42 min				
Parents	Parents from the purposive sample of 6 schools delivering AFLY5	*n* = 14 (one interview had two parents present so counted as two for number of participants but as one when calculating mean/range of interview time)	14 out of 18 (78 %)	October 2012 to April 2013	Semi structured interview questions on the following topics: **·**What contributes to a healthy lifestyle both generally and for children **·**Whether they were aware of their children taking part in healthy lifestyles lessons at school **·**Whether their child had bought home any homeworks or information relating to healthy lifestyles	Audio recording/transcript
		Face to face = 7/14 (50 %)				
		Mean length of interview = 39 min				
		Range = 28–52 min				
		Phone = 7/14 (50 %)				
		Mean length of interview = 40 min				
		Range = 23–53 min				

Copies of all the instruments used in the process evaluation can be found in the AFLY5 process evaluation plan [31]

### Ethics and consent

The study was approved by the Faculty of Medicine and Dentistry Committee for Ethics at the University of Bristol (reference number 111253). All adult participants provided written informed consent, while children gave written informed assent. (In England children under 16 cannot legally give consent so when they agree to participate in research it is described as providing assent) [32].

### Sampling

Phase 1: The five teacher training sessions, which involved all participating intervention schools, were observed. All participants were invited to provide structured feedback by questionnaire.

Phase 2: Observations of AFLY5 lessons being taught were carried out in all the intervention schools. The intention was to visit at least one class in every school at least once, and conduct two observations of each of the sixteen lessons being taught.

Phase 3: All teachers trained in delivery of AFLY5 were invited to participate in a semi-structured interview. Six intervention schools were purposively selected to ensure schools from localities with differing levels of area deprivation and with differing levels of teaching standards were represented. All study schools were defined as being in an area of high, medium or low deprivation by splitting them into thirds based on their score on the English Index of Multiple Deprivation 2010 (IMD 2010) and into having high or low levels of teaching quality defined by Office for Standards in Education (Ofsted) Scores [33]. (Ofsted is responsible for nationally assessing teaching quality in English schools and we used the schools’ Ofsted scores at the time of entry into the study in July 2011.) The Ofsted scores were Outstanding or Good (high teaching quality) and Satisfactory or Inadequate (low teaching quality). This process created 6 groups. Initially one school per group was randomly selected and approached for recruitment into Phase 3 of the study. If they declined or did not respond another was randomly selected. When recruitment slowed, all remaining schools were invited to participate on a first come first served basis. We aimed to interview two to three parents per school, and if more parents than required offered to take part in an interview, parents were selected to ensure a mix of: parent and child gender and children from across the Year 5 classes (if appropriate).

### Data collection

Details of all aspects of the data collected and the levels of response can be found in Table 2. In phase 1 all teachers and Learning Support Assistants who attended the training were asked to complete the training evaluation questionnaires. In phase 2 lesson observations were arranged at a convenient time for the teachers. Data collected during observations of the teacher training and lessons were largely qualitative as they mainly comprised the detailed notes written by the researcher describing what took place, with the researcher paying particular attention to specific topics such as level of engagement of those being trained or taught, questions asked and the suitability of the content for the ability of the group being taught. All teachers involved with the delivery of AFLY5 were asked to complete intervention delivery logs and return these to the research team. These logs sought information on, for example, who taught the lessons, whether or not they had been trained in the intervention, the date each lesson took place and how long it lasted. Teachers were also asked to record details of any amendments that they made. In phase 3 semi-structured interviews were conducted with Year 5 teachers in intervention schools. Topics included, for example, what they thought contributed to a healthy lifestyle, teaching health promotion in school and their experience of teaching AFLY5 lessons. Year 5 parents from each of the six intervention schools were invited to participate in an interview and asked what they thought contributed to a healthy lifestyle, whether they were aware of their children having been involved in any lessons at school about healthy lifestyles and if their children had brought home any homeworks on healthy lifestyles. With the exception of two of the five teacher training sessions that were observed by a colleague, all observations and interviews were undertaken by ER who was not involved in the AFLY5 intervention development, a point which was made clear to participating schools.

### Analysis

#### Data preparation

Data were extracted from the teacher training evaluation questionnaires and teacher intervention delivery logs and entered in an Access database. All handwritten teacher training and lesson observation notes were typed onto a structured pro-forma and interviews were digitally recorded and transcribed in full. Data from observations, interviews, and free text from the teacher logs and evaluation questionnaires were entered into NVivo10 (NVivo qualitative data analysis software; QSR International Pty Ltd. Version 10, 2012).

#### Data analysis

To assess data consistency when calculating the number of amendments made to lessons, a table was created to compare data from the teacher logs and lesson observations on a lesson by lesson basis. Descriptive summary statistics (means or medians, standard deviations or interquartile ranges and/or percentages) were calculated using an on treatment approach for the following quantitative variables: (i) training participants confidence teaching the nutritional lessons (ii) training participants confidence teaching the physical activity lessons (iii) lesson preparation time in minutes (iv) lesson preparation time the same as or different from usual (v) number of lessons delivered (vi) the number of children receiving a lesson (vii) number of weeks over which lessons were delivered (viii) number of teachers providing AFLY5 lessons in each school (ix) training status of those delivering AFLY5 lessons (x) whether or not lesson were amended and (xi) number of homeworks given out.

Qualitative data were analysed by ER in NVivo10 using a thematic approach [34]. Codes were generated both from the topics in the interview guides as well as iteratively from the data, initially discussed with RK, and were categorised as a series of themes. The themes were discussed, refined and agreed by ER, RJ, SW and RC. They are illustrated in this paper by selected, anonymised quotes which typify the data from interviews or text extracts from observation notes or teacher’s logs.

To maintain independence data analyses were undertaken by ER, SW, RJ and RC who were at that time blinded to the outcome of the main trial [35]. The analysts of the trial effectiveness data did not discuss results with anyone and submitted the final draft effectiveness paper to the chair of the AFLY5 steering committee. The analysts of this paper similarly submitted their final draft to the trial steering committee chair before seeing the results from the effectiveness paper.

## Results

Quantitative implementation fidelity findings on training and preparation, dose (number of lessons taught), reach (proportion of children receiving the lessons), and lesson amendments are presented below. The qualitative data, from interviews with 20 teachers (recruited from the pool of 29 intervention schools) and 14 parents (from the sample of six intervention schools), provide a more nuanced picture to contextualise the quantitative data. Quantitative and qualitative data on the modernising amendments made to the lessons in terms of length, differentiation for ability and increased engagement are presented before the final section which focuses on the teachers’ views of the intervention.

It was not possible to recruit a sample of six intervention schools for each of the six possible combinations of IMD and OFSTED score, however, there was an equal number of schools with high and low levels of teaching quality (three in each group) recruited, and two schools per IMD group (low, medium and high) were recruited.

### The extent to which the AFLY5 intervention was delivered as planned

#### Training and preparation

44 teachers from 29 schools delivered the intervention. 43 participants attended the training; 42 of these were teachers and 1 was a learning support assistant. Data from the teacher training evaluation questionnaires indicated that 42 of the 43 (98 %) teachers/teaching assistants who attended training agreed or strongly agreed with the statements “I feel confident that I can teach the nutrition sessions as per the lesson plans” and “I feel confident that I can teach the physical activity sessions as per the lesson plans”. Teacher interview data indicated that, on the whole, they appreciated having the opportunity to work through the programme during the training and, in particular, the opportunity to receive instruction on the physical activity component:

*I think the training we got when we came for the Active for Life was really, really helpful ‘cause it certainly pointed out a few things to us […] about like how easy it was to run different activities*

School 15, teacher 2 interview

*[…] I really liked the physical exercise training. And the activities that were supplied. Thought that was really good and gave me lots of ideas. I still use them, even though I’ve moved on to a different class[…] I thought the lady who ran the course was quite inspirational.*

School 50, teacher 3 interview

Using data reported by the 39 teachers who noted at least one lesson preparation time in the teacher log, the median length of lesson preparation time calculated across lessons delivered was 10 min (IQR 10–20). Data from 38 teacher logs relating to 450 lessons showed that for 47 % of the lessons teachers indicated that more preparation than usual was required, for 15 % less and for 38 % the same amount of preparation time as usual was required. During the interviews several teachers indicated negative feelings about the extra preparation time required and noted that it was often required for the physical activity sessions:

*I mean once the resources were made it was fine […] I don’t spend a lot of time preparing PE lessons. So I mean it might have been 15 min of reading through notes whilst it would have taken me 5 min normally so we are not talking a lot of time but […] if it takes you 15 min and you are teaching it for 45 min how much, how long does each lesson take to prepare?*

School 10, teacher 2 interview

#### Reach and dose

The reach, or percentage of children receiving all of the lessons taught, calculated from teacher log data, was 95 %. The timeframe over which the lessons were delivered was a median of 17.7 weeks (IQR 9.1–23.3). The teacher logs indicated that there were two main patterns of delivery: a) a regular dose, fairly evenly spread out; and b) a varied dose that changed in response to lack of time, curriculum or engagement issues. As one teacher explained during an interview, they delivered AFLY5 in a variable dose because of the length of time required to deliver the AFLY5 programme and the potential for diminishing engagement over time:

*.....it went over a term or well over one term, normally every term’s like a fresh start, something completely different […] so they need that chopping and changing ‘cause otherwise […] they’d hate it and that’s with anything, that’s not just with Active for Life.*

School 56, teacher 2 interview

41 of the 44 teacher logs were completed and returned to the study team. The remaining 3 teachers were contacted by telephone and asked to provide only the number of lessons delivered and (if possible) the dates of delivery. Data from these 44 teachers showed that the mean number of lessons delivered was 12.3 out of all 16 lessons (s.d. 3.7, median 13.5 lessons, range 1–16 lessons) which equates to 77 % of the intervention. The mean number of physical activity lessons delivered was very similar to that for nutrition lessons. Of the 41 teachers that returned teacher logs, and indicated that they had delivered some of the intervention, all of them delivered lesson 1, but delivery declined over the intervention period such that only 46 % delivered lesson 16. Seven teachers out of 41 (17 %) delivered all 16 of the lessons. The data from the teacher logs and interviews revealed that by far the most commonly mentioned reason for not delivering all the lessons was lack of time to fit all the lessons into an already full curriculum. This is explored further using data from the teacher interviews in the section ‘what amendments were made to the intervention’.

The mean number of homeworks delivered, calculated from data given in the teacher logs, was 6.2 out of a total of 10 (s.d. 2.6, range 2–10) equating to 62 %. Teachers who did not hand out the homeworks stated in both the teacher logs and interviews that they had to prioritise core skills homework above those from the intervention:

*All our homework is literacy + numeracy at the moment, building up to end of year tests.*

School 51, teacher 1, lesson 11- written extract from teacher’s log

The homeworks were designed to reinforce learning covered in the lessons and encourage parental involvement. In interviews with the parents five of 13 interviewed stated with certainty that their children had received AFLY5 lessons, and could remember homework items that were definitely part of the AFLY5 programme. Other parents were unsure about whether their children had received the lessons and homeworks.

#### Training status of those delivering the AFLY5 lessons

Of the 494 lessons with data on who delivered the lesson 386 (78 %) were delivered by someone who had received the training. Of the 108 sessions recorded as taken by staff not trained in the intervention, 25 (23 %) were covered by a main class teacher, 20 (19 %) were taken by Preparation, Planning and Assessment cover staff who enable teachers to work away from the classroom, 13 (12 %) were delivered by supply teachers, 9 (8 %) by student teachers, 9 (8 %) by teachers whose status was not recorded, 1 (1 %) by a learning support assistant and the remaining 31 sessions (29 %) were taken by people whose status was not recorded. AFLY5 lessons were seen as suitable to hand over, since the lesson plan, worksheets and homeworks were prepared. As data from the teacher logs and interviews revealed, the lessons that were handed over to these staff members, who were not trained in the use of AFLY5, were often the physical activity or physical education (PE) lessons

*I only taught a few lessons my PPA cover took the majority […] He did more the physical the activities, because he was taking PE*.

School 45, teacher 1, interview

### Amendments to the AFLY5 intervention

There was no guidance in the written materials provided to teachers about amending or adapting the lessons but they were told at the teacher training sessions that they should teach the lessons in the order that they were listed but that they could amend content as long as the message and learning outcomes remained the same. Observations of the teacher training sessions indicate that teachers were already considering, at that stage, how the lessons could be adapted.

*As they sit back down the teachers discuss how they might need to adapt a lesson for their own classes. One teacher is heard to say: “My kids can’t read so those work cards won’t work”*.

Observation notes, teacher training session on ‘Physical Activity games’ held on 27/09/2011

*The participants (teachers) engage well with Trainer 4, making comments or asking questions throughout the session about how particular activities might work in their classes or how they might adapt the games*.

Observation notes, teacher training session on ‘A Safe Workout’ held on 03/10/2011

Data from 39 teacher logs when cross referenced with 30 lesson observations, revealed that a total of 468 sessions had data showing whether or not the lesson was amended and that 28 % were amended. A majority (89 %) of the teachers amended the resources or lesson content on at least one occasion and each of the 16 lessons was amended by at least one of the teachers. Comparisons between lesson observations and teacher intervention delivery logs revealed that some teachers did not record amendments that were noted during the lesson observation. Of the 20 occasions where the teacher stated that they had not amended the session the observation indicated that amendments had been made in 9 (45 %) of these sessions.

### Reasons for amendments to the AFLY5 intervention

During interviews with 20 teachers from intervention schools (9 of whom were from schools included in the process evaluation), those who reported amending lessons said that they did so because they felt that the lessons or resource materials did not fully meet their needs. The reasons for their adaptations fell into four main categories: adjusting length of lessons to suit the overall ability level of a particular class; a need to differentiate for differing ability; conversion for use with new technology; and making the lessons more appealing to children to ensure their engagement.

#### Length of the lessons

The restrictions of fitting the lessons into the curriculum meant that lessons had to be altered according to the needs of children. However, the teacher’s perception of the children’s ability or interest in the lessons themselves also led to amendments to lesson length. As a teacher explained, it was a case of assessing their children’s needs almost on a lesson by lesson basis rather than applying the lessons as laid out in the plan:

*Just because when we looked at them, we go, there’s no way it’s going to take that long, I guess it’s knowing your children, knowing what to do. ........*

*And we realised that it wouldn’t, you know, what was a fifty minute lesson, you probably run that in half an hour*.

School 56, teacher 3

#### Differentiation to take account of ability

Amendments to the lessons and resource materials were also needed to differentiate for children with lower levels of ability, Special Education Needs or for whom English was an Additional Language (EAL). These amendments varied from class to class, although there were a reportedly large number of changes needed relating to the mathematical content, such as calculating the time spent on certain activities or the amount of sugar in certain drinks, as well as to the literacy content as some of the vocabulary was deemed to be too complicated. As this teacher explained:

*I did like what the ‘Active for Life’ was trying to do, it didn’t quite fit our curriculum really, and the materials were far too complicated […] because of the EAL issues.*

School 55, teacher 1 interview

#### Conversion of materials for use with new technologies

Amendments due to teaching style most often consisted of new slides that were compatible with interactive whiteboards. As one teacher explained when asked if they had made any amendments to the AFLY5 materials:

*We used Active Inspire* [interactive whiteboard]*[…] when we were teaching the lessons just to get it in a kind of format that we can use, just to make it a bit more user friendly*.

School 28, teacher 2 interview

#### Engagement

Several of the amendments under the category of engagement could also be seen as ‘user friendly’ changes, since they were primarily to make the lessons or resource materials more interesting, for either the teachers, the children or both. Amendments in this category included altering activities to include new aspects such as writing poems, making up raps, creating posters or doing role play. As one teacher explained, in relation to the nutrition lessons:

*I just changed them, made them more fun. They were really boring*.

School 50, teacher 3 interview

The idea that these lessons or materials needed to be made more ‘fun’ was mentioned in both the interviews and teacher logs, and was part of a theme identified in the qualitative data which indicated that teachers were unenthusiastic about the teaching materials in their original format because they felt that they were old fashioned.

### Teachers’ response to the intervention

While the quantitative fidelity of implementation data indicate that the AFLY5 intervention was well implemented, the interviews and teacher logs revealed a mixed view of the intervention. Teachers often noted the lack of time that they had to fit the lessons into an already full curriculum. This reasoning allowed teachers to present an acceptable ‘public’ explanation for not always implementing the intervention in full, which pointed to a structural constraint, and thus did not involve them in overt criticism of the intervention programme. There was a sense both during the interviews and in the analysis of the transcripts, however, that sometimes lack of time really meant lack of enthusiasm to make time or only to be fitted in when there was extra time. Teachers were not wholly negative or positive about the intervention; the vast majority of responses were mixed:

*So if anything this year we sort of almost missed it in a way because it was quite good at sort of, you know, filling, when we had little bits of time, pockets of time, we could, we could squeeze it in*.

School 50, teacher 1a interview

This did not mean, however, that teachers disliked the overall purpose of AFLY5, on the contrary they often mentioned how the messages behind the lessons were laudable but that there were presentational issues. As these teachers explained:

*It’s an amazing initiative, I think it was really, really important but it was just a huge amount to get through*.

School 56, teacher 2 interview

*So we did, a lot of the ideas were very good. But I just felt that the whole programme needs updating*.

School 51, teacher 2 interview

Problems or concerns with the resources provided as part of AFLY5 were mentioned by many of the teachers, either for not being suited to their class, as this teacher explains:

*Yeah I didn’t use any of your worksheets, I think I adapted every one of your worksheets*.

School 10, teacher 1 interview

or for being rather old- fashioned when compared to other available resources:

*I would suggest a DVD or website resource to support the learning[…] Although good, the resource does seem unambitious and rather old-fashioned*.

School 46, teacher 1, written extract from teacher’s log

The fact that teachers felt they had to alter the materials and that guidance and training on differentiation for ability was not provided as part of AFLY5 meant that there was a good deal of preparation for some teachers and this could also have contributed to the narrative regarding their lack of time. The results presented earlier revealed that 46 % of teachers felt that, on average, they needed more time to prepare the AFLY5 lessons compared to the regular lessons. This is perhaps not surprising given that these were completely new lessons. One limitation of this evaluation is that we did not determine how the preparation time for these lessons compared with that for any other completely new lesson. The trend towards more preparation time for PE lessons than nutrition lessons, for some of the teachers, could also reflect a general lack of enthusiasm for PE among some of the teachers. As this teacher reveals, this meant that when they were running out of time PE components were often dropped:

*And I have to admit if there are any bits that I skipped it was the PE bits because we were doing PE anyway, but those required more preparation for me than a normal PE lesson*.

School 10, teacher 1interview

This could be seen as part of a wider issue relating to the lack of training and lack of confidence in delivering PE experienced by some primary school teachers. As this teacher explains when describing why the AFLY5 training was so helpful:

*I am fairly keen on sport and PE in general but perhaps not the most confident in being able to teach it to children and stuff. So sort of taking it on board and being positive about it and seeing a sequence of lessons come about from it was actually very, very good*.

School 36, teacher 1 interview

Some schools have found that one way to address this problem was to employ dedicated staff responsible for delivering PE lessons across the school years. Teachers in some schools handed over all their PE lessons to these staff and AFLY5 PE lessons were no exception. Again there was a tendency towards some teachers to handing over the PE lessons in particular:

*I mean the handbook is quite straightforward and he is a bit of a sports, more of a sports expert so he brought his sports expertise to it and what he tended to do was, he’d do the Active for Life lesson and then he’d finish it up with a game or something so they actually had sort of like extra PE*.

School 15, teacher 2 interview

## Discussion

The data recorded in the teacher logs and observations of lessons presented in this paper show that AFLY5 was implemented with a good degree of fidelity. Reach was high as 95 % of children in intervention schools received lessons, 77 % of all the lessons were taught and 62 % of the homeworks were delivered. The average dose of 76.3 % of lessons compares favourably to similar school-based interventions utilising a curriculum based approach such as; *Project Tomato* with an average of 45 % of school lessons implemented [36], *Planet Health* which recorded over 70 % of lessons delivered at 5 out of 6 of the process evaluation schools [37], *Eat Well and Keep Moving*’s figure of 71 % of lessons delivered [38] and *HEALTHY PE*’s 87.6 % implementation rate [39].

Teachers did, however, record having to amend and adapt 28 % of the lessons and the observations suggested that teachers may have under-reported amending the lessons or had a different understanding of what constituted an amendment. While teachers voiced support for the aims of AFLY5 their views of the programme itself were more mixed. After their training in AFLY5, teachers recorded feeling confident that they could deliver the lessons, but when interviewed at the end of the intervention some reported reticence about delivering the lessons on physical activity, and a tendency to delegate this teaching to another colleague. These issues may mean that the intervention was not as well delivered as the teaching logs suggested, and that the AFLY5 intervention was less successful than it would have been had these issues been anticipated and dealt with. This accords with the effectiveness evaluation of AFLY5 which found that there was no difference in the primary outcomes of accelerometer measured physical activity, sedentary time or self-reported fruit and vegetable consumption among children in intervention compared to control schools, though there were beneficial effects with respect to reducing self-reported consumption of high energy drinks and snacks and screen-viewing time [27]. This indicates that while quantitative accounts of fidelity suggest that fidelity was good, more qualitative approaches are also needed to observe exactly what happens during the intervention delivery, and to explore the responsiveness of those involved in the delivery, if a more complete understanding of why an intervention is or is not effective is to be gained.

### Wider implications

Our findings have a number of implications for the development and evaluation of public health improvement interventions for use in educational settings. Firstly, the main reason for the omission of lessons or homeworks given by teachers in AFLY5 was a lack of time and pressure to focus on core literacy and numeracy skills. Finding the time to adapt the AFLY5 lessons for their children was also problematic for teachers. Educational policy in England and elsewhere increasingly emphasizes academic attainment and support for personal, social and health education has been downgraded since the feasibility study [40]. Evidence shows, however, that health and education are inextricably linked with the more educated enjoying better health and wellbeing, and students in good health having higher academic attainment [41]. Nevertheless, the primary purpose of schools is to educate, and those seeking to improve students’ health need to work closely with teachers to ensure that interventions are understood to be addressing both educational and health goals so that the time spent on health improvement interventions is not perceived as doing so at the expense of educational attainment. One way of demonstrating this is to include both health and educational outcomes measures in evaluations [42, 43]. AFLY5, like many other studies, did not do this [12], but this should be regarded as an essential requirement of trials of any future health improvement interventions in schools. Co-production of interventions by teachers, public health experts, parents and children is another way of achieving this and is likely to result in greater implementation fidelity. While co-production can be challenging [44] and we are unaware of any evidence that co-production provides superior outcomes than alternative approaches, this inclusive method of intervention development intuitively seems preferable to researchers designing and then implementing interventions. The Birmingham healthy Eating, Active lifestyle for Children Study (BEACHeS) is good example of a co-produced intervention [45], which showed evidence of promise in a pilot trial [46] and is now the subject of a definitive cluster RCT [47] in which many aspects of fidelity are being carefully documented.

Secondly, while most teachers endorsed the need to improve children's diets and increase levels of physical activity, some also expressed frustration with the lesson materials which they felt were out-of-date and too generic. Teachers were particularly frustrated by the work needed to adapt the lesson plans to make them suitable for children with different levels of ability and more interactive so that they could be taught using new technologies such as interactive whiteboards. This likely reflects the rapid change in use of teaching IT relative to the considerable time period currently required to develop an intervention and rigorously evaluate its effectiveness. Materials used in AFLY5 were developed originally in the USA in the late 1990s [48], developed in 2006 for the AFLY5 pilot and feasibility study which was undertaken during 2006 – 2009 [49]. Following an application for funding and further development work [50] the full-scale RCT began in 2011. This timescale highlights the need for a more flexible approach to designing and evaluating interventions and also the challenge in deciding how much to change an intervention which has been used successfully elsewhere. As suggested by Craig and colleagues pilot work should examine developmental uncertainties rather than simply being a small-scale version of the definitive trial [21]. There are already good examples of best practice when it comes to the recruitment and randomisation of schools in trials so that in future smaller scale piloting of the acceptability of intervention materials, perhaps integrated as an internal pilot stage of the main trial [51], would avoid intervention materials becoming out-dated and speed up the quest for effective public health improvement interventions.

Thirdly, our findings, like those of others [52], draw attention to the concerns that generalist schoolteachers have about teaching physical activity lessons. In our study some teachers said they valued the training AFLY5 provided on this, however, these lessons were more likely to be delegated to other staff who had not been trained in the AFLY5 intervention. Acknowledging this issue when designing physical activity lessons, and ensuring that all those likely to get involved in the delivery of such an intervention are trained in it would help to ensure that fidelity is maintained.

### Limitations

The proportion of teachers who provided data and the amount of data provided by them varied considerably across schools. In the case of teacher logs, none were fully completed therefore they only provided a partial picture of what happened during the AFLY5 lessons. Again, this has implications for the design of future trials in schools as comprehensive data collection also adds to the time teaching staff have to spend on something that may not be perceived as central to their job. There was potential for bias if only those who felt particularly strongly about either the intervention, or the research process itself, agreed to take part in interviews. However, as the majority of data considered in this paper came from all of the intervention schools in the trial, and a range of views were offered by teachers and parents, it seems unlikely that such a bias has influenced our findings.

Recruitment targets for parent interviews were based on previous research and were met in all but one of the intervention schools in the process evaluation. The recruitment process itself, however, was lengthy and both the parent and teacher interviews were carried out after the intervention finished (median was 288 days after the intervention ended). This could account for the lack of detail and recall in parental accounts and in some teacher accounts. In addition, the lack of detail around AFLY5 homeworks in parental accounts may also be due to the fact that despite the homeworks being designed to ensure the AFLY5 messages made it home to families, and some required parents to assist in their completion, the intervention was designed to fit in with the current curriculum and so AFLY5 homeworks may not have been easily distinguished from other homeworks.

### Strengths

The major strength of this study is the use of multiple sources of data which has allowed us to cross check information reported on the same issue. This more detailed information has enabled us to build a more complete picture of how the intervention was delivered and received. This nuanced account of how and why the teachers adapted the intervention materials would have been difficult to achieve from the data recorded in the teacher logs alone or by using questionnaires. Thus, this paper highlights the value of incorporating qualitative research methods into process evaluation. A key strength of this study is that the analyses of data were conducted with no knowledge of the effectiveness of the intervention itself. This means that our conclusions regarding fidelity of the intervention’s implementation were not influenced by knowing whether or not the intervention actually worked, or vice versa.

## Conclusions

While the fidelity of implementation in terms of quantity of lessons and homeworks delivered was good, the difficulties of incorporating some of the AFLY5 materials into more technologically advanced and interactive current teaching practice, coupled with pressure on teachers’ time, and a need to adapt the materials to suit students’ differing abilities and ensure their engagement resulted in mixed enthusiasm for AFLY5. This, together with a tendency to delegate teaching of physical activity lessons to those not trained in the intervention, may have meant that the intervention messages were not as successfully delivered as anticipated and explain why the intervention was found not to be effective.
